# Commentary: Kappen S, Jürgens V, Freitag MH, Winter A. Attitudes Toward and Use of Prostate-Specific Antigen Testing Among Urologists and General Practitioners in Germany: A Survey

**DOI:** 10.3389/fonc.2022.841858

**Published:** 2022-01-20

**Authors:** Kay-Patrick Braun, Ingmar Wolff, Steffen Lebentrau, Matthias May

**Affiliations:** ^1^ Medizinisches Versorgungszentrum (MVZ) Dr. Braun GmbH, Cottbus, Germany; ^2^ Department of Urology, University Medicine Greifswald, Greifswald, Germany; ^3^ Department of Urology, Werner Forssmann Clinic, Eberswalde, Germany; ^4^ Department of Urology, St. Elisabeth Hospital Straubing, Brothers of Mercy Hospital, Straubing, Germany

**Keywords:** prostatic neoplasms, early detection of cancer, prostate-specific antigen, physicians, healthcare surveys, attitudes, guideline adherence

## Introduction

The widespread use of Prostate-specific antigen (PSA)-based early detection (opportunistic PSA-based screening) of prostate cancer (PCa) since the beginning of this century has led to a marked reduction in cancer-specific mortality (CSM) and a simultaneous increase in incidence (1). However, screening also gave rise to an increasing detection of low-risk PCa without immediate need for treatment, resulting in controversial and critical discussions on the usefulness of opportunistic PSA-based screening which continue to the present day (1, 2). The US Preventive Task Force issued a recommendation against PSA screening in 2012, which was revised in 2017 as a consequence of the observed stage shift towards more advanced and metastatic stages resulting from the initial recommendation, in addition to study data supporting the evidence that PSA-based screening results in a significant reduction of CSM (1, 3). Accordingly, it is now indisputable that PSA screening results in a decrease in CSM (by 35% after 18 years of follow-up in the Göteborg arm of the European Randomized study of Screening for Prostate Cancer) and a lower incidence rate of metastatic disease stages, but it is also associated with a substantial risk of overdiagnosis (1, 4). False-positive findings and resulting eventual complications of prostate biopsy as well as subsequent overtreatment represent potential harm to patients invited for PSA-based screening (5).

Worldwide, opportunistic PSA-based screening for PCa is performed by different professional groups potentially resulting in qualitative differences. In addition, the introduction of innovative strategies for PSA-based early PCa detection (risk-adapted approach, integration of multiparametric magnetic resonance imaging) poses new challenges for physicians involved (1, 6, 7). Meritoriously, the working group around Sanny Kappen analyzed the attitudes of dominant professional groups responsible for opportunistic PSA-based screening in Germany (namely general practitioners (GPs) and urologists) using a comprehensive survey (5, 8–13). With great interest we noticed their recently published results which we would like to comment (11).

## Discussion

For colleagues from countries in which PSA-based early detection of PCa is at least in part provided by non-urology specialties, Kappen et al. present extremely important data (11). In Germany (as, for example, in the US), GPs perform a large part of opportunistic PSA-based screening (5, 8–13). Despite the selectivity of results (preconceiving the considerable bias resulting of GPs’ response rate of only 6.1%; query was performed in one German state only), the study by Kappen et al. provides answers to the following important questions: 1) What is the proportion of GPs performing PSA testing?, 2) What expectations do GPs associate with opportunistic PSA-based screening?, 3) Are current studies and guideline recommendations known and are they implemented in daily routine (especially concerning the target population of opportunistic PSA-based screening)?, 4) What quality of patient counselling precedes PSA determination?, and 5) What consequences are drawn from pathologically elevated PSA levels? (Or: At which point in time do GPs schedule the integration of the urologist?).

**Table 1** compares results on selected items from the current and another study by Kappen et al. with our own results obtained some years earlier within a German population of GPs and internists (8, 9, 11, 12). The willingness of non-urologists to perform PSA-based early detection of PCa in asymptomatic men has decreased over the years (83.9% vs. 51.2% and 55.2%, respectively, both tests with p<0.001; **Table 1**). In this context, it seems noteworthy, that no significant difference was observed regarding this point between the first study by Kappen et al., which was smaller in terms of the number of GPs included, versus the current study by Kappen et al. comprising a higher number of cases (51.2% vs. 55.2%, p=0.711). Comparing their earlier 2016 study with our study from 2012, Kappen et al. found a significantly higher proportion of primary care physicians who did not perform PSA-based early detection of PCa at all in asymptomatic men (p<0.001; **Table 1**). However, a significantly higher rate of non-urologists considered the reduction of PCa-specific mortality based on PSA screening as scientifically proven in the recent study by Kappen et al. compared to our data (20.8 vs. 12%, p=0.030). In contrast, we found this to be inconsistent with the reported higher screening readiness of non-urologists in their first study (12). In our opinion, it seems very important that Kappen et al. showed that patients with elevated PSA levels were significantly less likely to be directly referred to a urologist compared to our own results (53.1 vs. 68.6%, p=0.006). This hesitation may result in a delay in PCa diagnosis, possibly hampering patients’ prognosis.

**Table 1:** Comparison of selected items from all studies conducted in Germany to survey non-urological physicians regarding their attitudes towards PSA-based early detection of PCa.

Characterization of the studies	[11]	[12]	[8,9]	p*	p**
Year of the questionnaire study	2019	2016	2012	*-*	*-*
Size of the primary study group contacted	1579	172	600	*-*	*-*
Specialty of the non-urological physicians contacted	All GP	All GP	385 GP and 215 internists	*-*	*-*
Returned questionnaires	96	47	392	*-*	*-*
Size of the final evaluable study group	96	41	392	*-*	*-*
Resulting response rate	6.1%	23.8%	65.3%	*-*	*-*
**Presentation of selected items**					
Proportion of physicians who recommend PSA-based ED of PCa to asymptomatic patients	55.2% (n=53)	51.2% (n=21)	83.9% (n=329)	*<.001*	*<.001*
Proportion of physicians who would not recommend PSA-based ED of PCa at all to asymptomatic patients	n.a.	39.0% (n=16)	10.2% (n=40)	*n.a.*	*<.001*
Proportion of physicians who consider the reduction of PCa mortality by PSA screening to be proven	20.8% (n=20)	n.a.	12.0% (n=47)	*.030*	*n.a.*
Proportion of physicians who primarily transfer patients with pathologically elevated PSA-levels to urologists	53.1% (n=51)	68.3% (n=28)	68.6% (n=269)	*.006*	*1.000*

p*, statistical difference calculated using the Chi² test (Fisher´s exact test, two-sided) between the study of Kappen et al. (11) and our own study [8,9]; p**, statistical difference calculated using the Chi² test (Fisher´s exact test, two-sided) between the study of Kappen et al. (12) and our own study [8,9]; ED, early detection; GP, general practitioners; n.a., not available; PCa, prostate cancer; PSA, prostate-specific antigen.

A tiny downer in the otherwise excellent work of Kappen et al. is certainly that the authors solely opted for a purely descriptive analysis of their data (11). Considering multivariate analysis of our own survey study, it was striking that non-urologists who never attended topic-specific education by urological colleagues were almost 4 times more likely to perform opportunistic PSA-based screening of patients (compared with those who attended urological education events; OR 3.95, p=0.002) (9). Thus, a frequently observed phenomenon of medical practice seems to be confirmed: The more intensively one approaches the crucial aspects of a medical problem, the greater the humility in front of the complexity of the underlying issue gets.

Another remarkably interesting point in the work of Kappen et al. is the question of knowledge of the interdisciplinary S3 guideline led by the German Society of Urology (DGU) and the recommendations of the German Society of General Medicine (DEGAM) (13, 14). This fact is of utmost importance as statements differ considerably. While the DEGAM recommendation, which is three years older, advocates PSA-based early detection for PCa only for those patients who actively request it, the interdisciplinary S3 guideline allows physician’s active initiative, provided that the patient is thoroughly informed about possible advantages and disadvantages of this PCa screening measure (13, 14). This could partly explain different attitudes of GPs and urologists towards PSA-based early detection.

We are currently conducting a study involving 150 GPs enabling us to analyze their attitude and individual approach towards opportunistic PSA screening (KABOT study, Knowledge And Belief Over Time). Additionally, in this study, 50 consecutive male patients aged 45-70 years will receive a questionnaire from each participating GP. This allows, among other things, to analyze the type and extent of previous PSA testing based on patient’s reports. Thus, after analysis of the KABOT study data, we should be able to answer the five questions above even more sufficiently.

## Author Contributions

All authors listed have made a substantial, direct, and intellectual contribution to the work and approved it for publication.

## Funding

Open access publication was funded by the MVZ Dr. Braun GmbH.

## Conflict of Interest

The authors declare that the research was conducted in the absence of any commercial or financial relationships that could be construed as a potential conflict of interest.

## Publisher’s Note

All claims expressed in this article are solely those of the authors and do not necessarily represent those of their affiliated organizations, or those of the publisher, the editors and the reviewers. Any product that may be evaluated in this article, or claim that may be made by its manufacturer, is not guaranteed or endorsed by the publisher.
